# Fluoxetine attenuates apoptosis in early brain injury after subarachnoid hemorrhage through Notch1/ASK1/p38 MAPK signaling pathway

**DOI:** 10.1080/21655979.2022.2037227

**Published:** 2022-04-06

**Authors:** Ming Liu, Weiying Zhong, Chao Li, Wandong Su

**Affiliations:** Department of Neurosurgery, Qilu Hospital of Shandong University, Jinan City, Shandong Province, China

**Keywords:** Apoptosis, fluoxetine, subarachnoid hemorrhage (SAH), serotonin selective reuptake inhibitor (SSRI), Notch1

## Abstract

Subarachnoid hemorrhage (SAH) is a severe brain condition associated with a significantly high incidence and mortality. As a consequence of SAH, early brain injury (EBI) may contribute to poor SAH patient outcomes. Apoptosis is a signaling pathway contributing to post-SAH early brain injury and the diagnosis of the disease. Fluoxetine is a well-studied serotonin selective reuptake inhibitor (SSRI). However, its role in apoptosis has not been clearly understood. The present investigation assessed the effects of Fluoxetine in apoptosis and the potential Notch1/ASK1/p38 MAPK signaling pathway in EBI after SAH. Adult C57BL/6 J mice were subjected to SAH. Study mice (56) were randomly divided into 4 groups: the surgery without SAH (sham (n = 8), SAH+ vehicle; (SAH+V) (n = 16), surgery+ Fluoxetine (Fluox), (n = 16) and SAH+ Fluoxetine (n = 16). Various parameters were investigated 12, 24, 48, and 72 h after induction of SAH. Western blot analysis, terminal deoxynucleotidyl transferase-mediated deoxyuridine triphosphate nick-end labeling (TUNEL) staining, Immunohistochemistry (IHC), and flow cytometry were carried out in every experimental group. According to the findings, the SAH downregulated NOTCH1 signaling pathway, Jlk6 inhibited Notch1, Notch1 inactivation increased apoptotic protein expression and suppressed Bax, and cytochrome C. Fluoxetine reversed the effects of notch1 inhibition in SAH. The Neuroprotective Fluoxetine effects involved suppression of apoptosis post-SAH. In summary, early Fluoxetine treatment significantly attenuates apoptosis and the expression of apoptosis-related proteins after 72 h post-SAH. Fluoxetine may ameliorate early brain injury after subarachnoid hemorrhage through anti-apoptotic effects and Notch1/ASK1/p38 MAPK signaling pathway.

## Graphical Abstract

**Figure:**
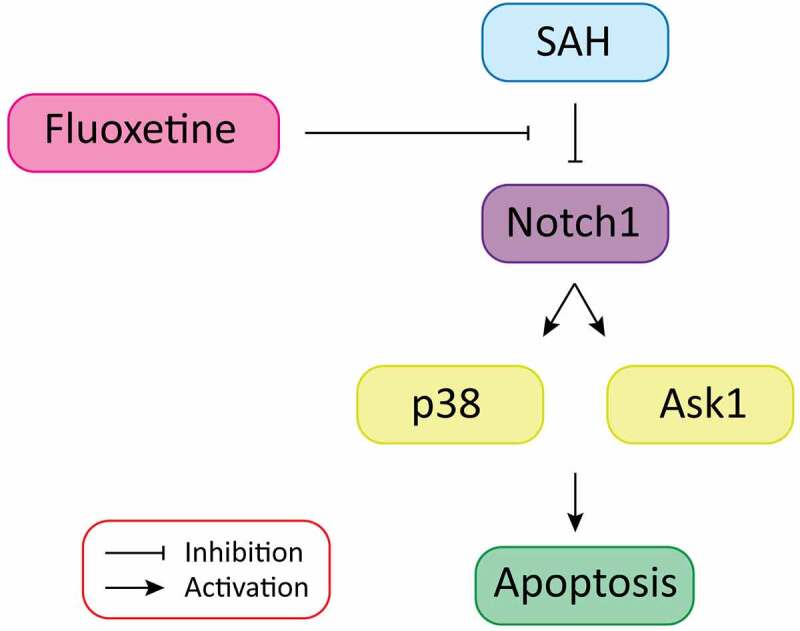

## 1. Introduction

Subarachnoid hemorrhage (SAH) is a severe brain condition associated with a significantly high incidence and mortality [1]. Early brain injury (EBI) refers to the immediate injury to the whole brain, which involve death of brain cell, disruption of blood–brain barrier (BBB), brain edema, and microvascular malfunction within the first 72 h of post-onset [2]. According to investigations, EBI emanates from SAH, and may contribute to poor patient SAH outcomes. The possible mechanisms in SAH involve upregulated intracranial pressure, a reducing cerebral flow of blood, disruption of the BBB, and brain swelling [3]. Regardless of the recent developments in endovascular surgical and microsurgical techniques, the SAH patients’ outcomes remain low [4]. The poor recovery rate mandates the new therapeutic drugs development, especially endogenous remedies with increased advantages regarding efficacy, safety, affordability, and tolerability [5].

Apoptosis is among the various cellular processes in SAH [6], and it has been shown to lead to cell death after SAH [7]. The process contributes immensely in post-SAH early brain injury and to the diagnosis of the disease [8]. In case of a ruptured aneurysm, an upregulated intracranial pressure, ischemic-elevated radicals and toxic components discharged from the blood leads to apoptosis of the neurons [9]. Thus, therapeutic targets of apoptosis signaling may offer protection against SAH [10].

Notch1 signaling pathway is essential in the regulation of cell fate decisions and in homeostasis of tissues [11]. On attaching to Jagged-1 or its ligands Delta-1, Notch1 receptor get cleaved using disintegrin-metalloprotease tumor necrosis factor (TNF)-α-converting enzyme and the γ-secretase complex, which generates the Notch1 intracellular receptor domain (NICD) that comes from the membrane [11]. NICD subsequently translocates to the nucleus, thus acting with the transcription factor recombining binding protein suppressor of hairless (RBP-Jκ), together with other modulators to regulate various cellular metabolisms, as cell differentiation and proliferation [11]. Additionally, Notch1 signaling has been shown to play important function in activating the inflammatory response in different diseases. Recent investigations showed that Notch1 signaling contributed to the activation of microglia-mediated neuroinflammation [12] and increased degeneration of neurones, through nuclear factor-κB (NF-κB) signaling pathway in CNS infections [11].

Fluoxetine is a well-studied serotonin selective reuptake inhibitor (SSRI) [13]. Because of its minimal toxicity and side effects, Fluoxetine prescription has been made mostly against anxiety disorders and depression [14]. Fluoxetine neuroprotective roles have been shown in various neurological conditions [15,16]. Investigations have confirmed its ability to modulate neuroinflammation [17] and to inhibit the activation of TLR4 and NF-κB pathways [18]. Further, reports have confirmed the ability of Fluoxetine to inhibit activation of NLRP3 inflammasome and its subsequent capability of causing cell death by necrosis in EBI after SAH [19]. The p38 mitogen-activated protein kinase (p38 MAPK) is essential in responding to various extracellular stresses responsible for various physiological processes, including cellular proliferation, survival, inflammation, and apoptosis [20]. However, the possible role of Fluoxetine in apoptosis remains unclear.

The present investigation hypothesized that Fluoxetine suppresses apoptosis through Notch1/ASK1/p38 MAPK signaling pathway in EBI after SAH. The aims of this investigation was to determine the effect of SAH on Notch1 signaling pathway, investigate the role of Jlk6 on Notch1, to elucidate the effects of Notch1 inactivation on apoptotic proteins expression, and to investigate the role of Fluoxetine on SAH-induced neurologic function impairment. The study also aimed at assessing the effects of Fluoxetine on notch1 inhibition in SAH, and finally to study the mechanisms of neuroprotective Fluoxetine effects after SAH.

## 2. Materials and methods

### 2.1 Experimental animals and study design

Male C57BL/6 J mice (25–30 g, 8–10 weeks old) were purchased from our institutional animal center. The mice were housed in cages that had free water and food access at 24°C ± 2°C and 60% ± 5% humidity with a 12-h light/dark cycle. Mice were fed ad libitum with standard chow and sterilized tap water. All the surgical procedures were approved by our institutional Ethics Committee. Fifty six (56) mice were randomly divided into a sham group; surgery without SAH induction (n = 8), SAH+ Vehicle; (SAH+V) group (n = 16), surgery + Fluoxetine group, (Fluox) (n = 16) and SAH+ Fluoxetine group (n = 16).

### 2.2 Induction of SAH mice model

To induce brain injury, we used the SAH method and killed animal models at different intervals, as used previously [21]. Summarily, mice were anesthetized with pentobarbital (50 mg/kg). The left-carotid artery, together with its branches were opened and subsequently separated. Later, an excision of the left external carotid artery (ECA) was made. The procedure was followed by a monofilament suture (4–0), which was advanced through the internal carotid artery (ICA) via ECA till a resistance feeling was obtained. The suture was finally inserted more with the aim of puncturing the vessel and inducing SAH. SAH was induced in 48 animals, randomly divided into 3 groups (n = 16), as mentioned in the study design. First, animals in each SAH group were killed at 12 h (n = 4), 24 h (n = 4), 48 h (n = 4), and 72 h (n = 4) after induction of SAH. Samples were then collected for subsequent assays. In the next experiment, Fluoxetine was delivered in the animal model. Fluoxetine (Sigma-Aldrich #56,296-78-7) was dissolved using sterile 0.9% sodium chloride. A solution of 10 mg/kg of Fluoxetine or vehicle was intravenously injected 6 hours following the introduction of SAH [22]. Equal amount of 0.9% sodium chloride was intravenously administered to the sham and vehicle mice at similar time points after injury.

### 2.3 Cells culture

In vitro study was done using adherent glioblastoma (GBM) brain cell lines. The GBM cells were grown in culture plates in Dulbecco’s modified Eagle’s medium DMEM, supplemented with 10% fetal bovine serum, 100 U/ml penicillin, and 100 µg/ml streptomycin. The growth and maintenance condition was at 37°C, and 5% CO_2_. Splitting of cells was done at the exponential phase when 70–80% confluence had been achieved. The cells were rinsed in PBS, digested with 0.25% trypsin and EDTA solution, and centrifuged at 1000 rpm for 5 minutes. The cells were subsequently inoculated into new culture plates.

### 2.4 Cell transfection

The brain cells ‘Human GBM cell line’ were grown for 48 hours before transfection. The transfection procedure was done at a confluence of 70%. A plasmid (1-µg) aliquot (Notch1 OE or si-Notch1 – Genechem, Shanghai, China) was dissolved in a medium free of serum. A transfection reagent (Vegofect 1:1,000; Vigorous Biotechnology Co., Beijing, China) was dissolved in the medium free of serum as per the manufacturer’s guidelines. The transfection solution and oligonucleotides were mixed then incubated for 15 minutes without stirring. The resulting mixture was later added dropwise to the serum-free culture medium. Slight shaking of the culture dish was done for an even distribution of the mix. For Notch1 silencing, the brain cells were transfected with small interfering RNA targeting Notch1 (si-Notch1 or the si-NC). For Notch1 inhibition, brain cells were incubated with JLK6 (7-amino-4-chloro-3-methoxyisocoumarin) prepared from a stock solution of 10 mM in dimethylsulfoxide (DMSO). In brief, 1 µM of JLK6 was added to the serum-free culture medium. The cells were then cultured for 24 hrs.

### 2.5 Reverse transcription-quantitative polymerase chain reaction (RT-qPCR)

Extraction of Total RNA was done using TRIzol (Life Technologies, Carlsbad, CA, USA). According to the manufacturer’s guidelines, cDNA was then synthesized via reverse transcription using TaKaRa Reverse Transcription Kit. The cDNA template (1 μL) was prepared with 0.4 μL RT-FP (10 μmol/L), 0.4 μL RT-RP (10 μmol/L), 8.2 μL RNA-free enzyme water, and 10 μL 2 × SYBR Green Mix. The relative mRNA expression was determined after 40 cycles using the 2^−ΔΔCt^ method [23]. The primers used were obtained from Huada Gene Co. (Shenzhen, China). U6 and GAPDH were used as internal control. The sequence of primers used are presented in Table 1.

**Table 1.** Vaccination knowledge score by different socio-demographic and work-related characteristics of participants

Genes	Primer sequences (5’- 3’)
Hes1	Forward: GCCCCCAGGATTGACCTCTG
	Reverse: TGCAGCACAAAGTCTAACGCC
Hes5	Forward: GAAGCACAGCAAAGCCTTCGT
	Reverse: AGGCACCACGAGTAGCCTTC
Jag1	Forward: ATCGAGTGCCGCATCTCACA
	Reverse: GCTGCAAGGGGACACACAAC
Notch1	Forward: GGGCCACCCCTCCTAGTTTG
	Reverse: CCGAGCTGAGCCAAGTCTGA
U6	Forward: CTCGCTTCGGCAGCACA
	Reverse: AACGCTTCACGAATTTGCGT
GAPDH	Forward: AGAAGGCTGGGGCTCATTTG
	Reverse: AGGGGCCATCCACAGTCTTC

### 2.6 Immunohistochemistry

Frozen sections of the brain (5 µm) were cut into sterile glass slides, air-dried, then fixed for 10 minutes in chilled acetone at −20 degrees [24]. Firstly, the slides were washed using tris-buffered saline (TBS) then incubated using less than 1% H_2_O_2_ in methanol. Next, the slides were exposed to three washes in distilled water. Later, the washed sections were blocked using 10% of normal rabbit serum. The frozen sections were then fixed for 20 minutes using cold acetone. Nonspecific labeling blocking was done by incubating the sections with 5% bovine serum albumin, which was diluted for 30 minutes in PBS before the addition of antibodies. Primary antibodies including anti-Notch1 (ab8925), anti-Hes5 (ab194111), anti-Hes1 (ab71559), and anti-Jag5 (ab109536), were then added, and the samples were incubated for 24 h at 4°C. Later, corresponding secondary antibodies were added and incubated for 90 minutes, washed thrice for 10 minutes in PBS and thrice in PBST. Finally, the brain sections were incubated using rhodamine-conjugated sheep anti-rabbit or FITC-conjugated sheep anti-mouse secondary antibody diluted to 1:200 using 5% BSA fraction in 0.1% PBST at a room temperature for 2 hours in the dark. Finally, the samples were washed three times in PBS. All the brain sections were incubated with 1 μg/mL of 4’,6-diami-dino-2-phenylindole, and 2 μg/mL of propidium iodide for counterstaining. Tissues were then observed under a confocal microscope (Zeiss, Germany).

### 2.7 TUNEL staining assay

TUNEL staining was done as described elsewhere [25]. The experimental animals were sacrificed with deep anesthesia through perfusion (cardiac) using chilled PBS and 4% paraformaldehyde (30,525–89-4, Sigma-Aldrich) (both of pH 7.4). Later, brain fixing was done by immersing the brain in 4% paraformaldehyde overnight at 4°C. The sample was then transferred into sucrose solution (30%) to ensure the brain sank deep at the solution’s bottom. The sample was later frozen in tissue-freezing media. The brain’s cortical sections were then cut to a dimension of 7 mm. The TUNEL staining experiment was then performed as per the manufacturer’s (Roche, Basel, Switzerland) instructions. Briefly, brain-sectioned samples were placed on glass slides. Incubation was then done using a mixture of TUNEL reaction, which included a solution of an enzyme (terminal deoxynucleotidyl transferase) together with FITC-labeled TUNEL-positive nucleotides placed in a humidified chamber (in the dark) at 37°C for 1 hr. The samples were then washed for three times in PBS for 3, 9, and 5 minutes, respectively. They were later covered using water-based mounting media. The images were then captured by a BX51 light microscope (Olympus). The number of cells positive for TUNEL that demonstrated green stained nucleus and all the cells which stained the nuclear blue DAPI (40, 6-diamino-2-phenylindole) were determined through a random count of five fields through high power magnification. The apoptosis index expression was shown as the ratio of cells that stained positive for apoptosis to the total cell number counted x 100%.

### 2.8 Flow cytometry

For the detection of apoptosis, cultured brain cells were harvested post-treatment. The cells were prepared under the apoptosis detection kit’s instructions (BD, CA, USA). In summary, cells incubation was done using Annexin V-FITC solution (A13199, ThermoFisher) (5 μl) at 4°C for 15 min and PI dye solution (10 μl) at room temperature for 5 min. The cells were then analyzed through flow cytometry. The cells which stained positive for both Annexin V-FITC and PI+ and those which were single positive for Annexin V-FITC but negative for PI-cells were regarded as apoptotic cells.

### 2.9 Neuro-behavioral tests

A blinded observation of the Neurological tests was done on all of the mice at 24 and 72 h post-SAH. Assessment of enduring behavioral deficits were done using locomotor activity, rotarod test, tapered beam walking, beam balance, and grip strength test. Presentation of the daily session data were done as mean ±SEM of three consecutive experimental trials. To determine the locomotor activity, mice were placed in a transparent chamber of Plexiglas (41 × 41 × 30 cm) inside an Optovarimax (Med Associates, Inc., USA) for about 10 min to record the behavior [26]. The animals were placed individually in the middle of the chamber and free ambulation allowed for 10 min. The vertical beams’ numbers broken was used to indicate vertical rearing, whereas the traveled horizontal distance was recorded to indicate ambulatory activity. Locomotor activity was the total sum of vertical rearing and ambulatory activity. Assessment of SAH-induced sensori-motor deficit was carried out through accelerating rotarod test (Ugo Basile Biological Research Apparatus, Italy). Mice were trained every day for 5 days through an application of a schedule. The mice were trained on staying on a rotarod accelerating at 4–40 rpm for over 5 minutes, with rising steps of 4 rpm at intervals of 30s for three trials daily. The fall latency’s average was recorded [27]. Tapered beam walking was a learned avoidance test with similarity to the test utilized by Silasi and Colbourne [28] with some minor modification. In brief, mice were trained in traversing a tapered beam (1.6-m long and 6 cm wide at the start) and evenly tapered to a final width of 1.5 cm from the noise and light source. A piece of wood, 2 cm wider than the beam, was attached to the under surface of the beam to act as a ledge used by the mice in stepping down in case of failure to balance on the beam’s top surface. Normal mice were capable of traversing the greater length of the beam without stepping down onto the ledge, while the set-up encouraged the injured mice to demonstrate their deficits through stepping down onto the ledge’s side contralateral to an injury. The mice were trained for five days (3–5 trials daily) after which the performance at baseline was video recorded. A mirror was placed behind the beam to allow for the simultaneous filming of both sides of the mice, using a single camera. The percentage of impaired steps with the contralateral hind paw, whose definition was the limb that make contact with the supportive ledge, was then recorded for every session of testing. The test of a beam balance required mice to balance on a raised wooden beam; 60-cm square (1.0 cm wide and 50 cm above the floor). The mice were later perpendicularly positioned on the beam’s center for 60s maximum [29]. Grip strength in all animals was determined for assessment of neuromuscular strength [30]. Summarily, the mice were let to hold the grid, which was later pulled back horizontally until the mice released their grip. Finally, this observation was recorded as its length.

### 2.10 Western blot

Western blotting was done following the previously published guidelines [31]. Summarily, the tissue of the brain was homogenized and then spun. The protein content was then determined by a detergent-compatible protein assay kit (Bio-Rad, California, USA). Equal protein amounts (approximately 60 mg) were then loaded into the sodium dodecyl sulfate-polyacrylamide gel electrophoresis (SDS-PAGE) wells. The separated protein samples were then transferred to a nitrocellulose membrane after electrophoresis. Blocking of the membrane was then done for 2 hours using a buffer (nonfat dry milk), followed by overnight incubation at 4°C using anti-Notch1 (ab8925), Hes 1 (ab71559), Hes5 (ab194111), Jag 1(ab109536), Caspase 3 (ab32351), BCl-2 (ab182858), Cytochrome C (ab133504), Bax (ab32503), P-ASK1 (#3765, Cell Signaling), T-ASK1 (#3762, Cell Signaling), P-p38 (sc-166,181),T-p38 (sc-7972), and β-actin (sc-47,778) primary antibodies. Later, membranes were then incubated with secondary antibodies (conjugated with horseradish peroxidase) for one hour at room temperature. The resulting membranes were finally exposed to the x-ray film, and the densities of the band were assessed with Image J software.

### 2.11 Data analysis

Data were analyzed using Graph Pad Prism 6 (Graph-Pad Software Inc., CA, USA). Presentation of values was done as the mean ± standard error of the mean (S.E.M.). Comparisons of the groups were made using ANOVA. In facilitating comparisons between the various groups, band density values in Western blot analysis were normalized to the control group mean value. Values were regarded as significant when P < 0.05.

## 3. Results

### 3.1 SAH downregulates Notch1 signaling pathway

Subarachnoid hemorrhage (SAH) is a severe brain condition associated with a significantly high incidence and mortality. Fluoxetine is a serotonin selective reuptake inhibitor (SSRI) whose role in apoptosis has not been clearly understood. The present investigation hypothesized that Fluoxetine suppresses apoptosis through Notch1/ASK1/p38 MAPK signaling pathway in EBI after SAH. The aims of this investigation were to determine the effect of SAH on Notch1 signaling pathway, investigate the role of Jlk6 on Notch1, to elucidate the effects of Notch1 inactivation on apoptotic proteins expression, and to investigate the role of Fluoxetine on SAH-induced neurologic function impairment. The study also aimed at assessing the effects of Fluoxetine on notch1 inhibition in SAH, and finally to study the mechanisms of neuroprotective Fluoxetine effects after SAH.

**Figure 1.**
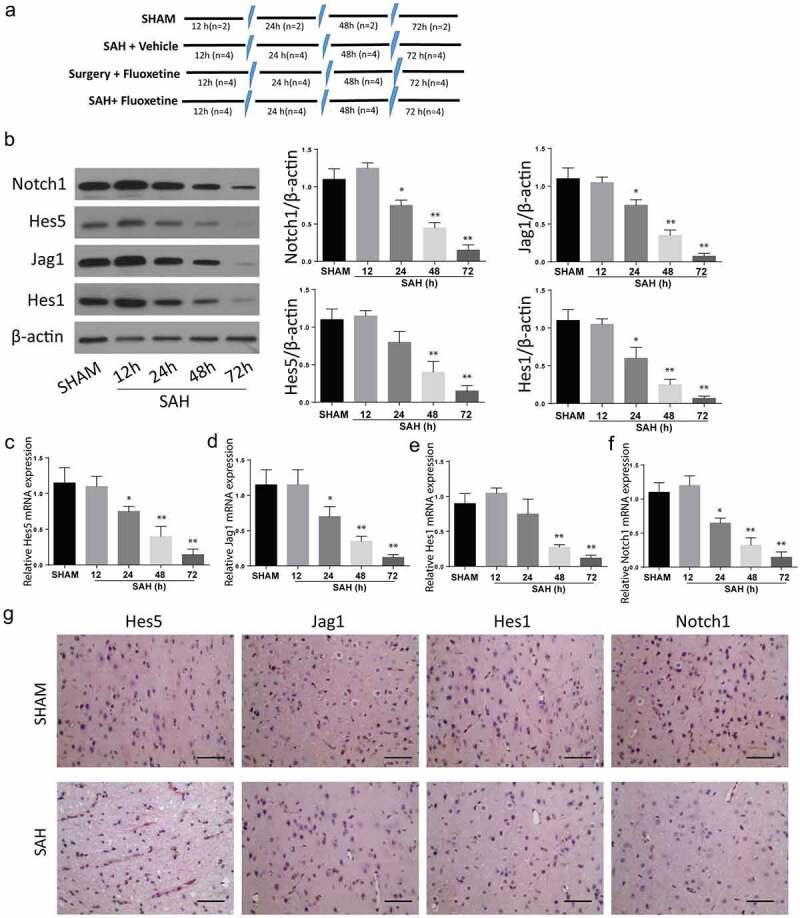
**SAH-induced brain injury impairs Notch1 signaling**. A: Schematic representation of animal grouping used in this study. B: Western blot analysis exhibiting protein expression of Notch1, Hes1, Jag1 and Hes5. B-E: RT-qPCR analysis of Hes5 (c), Jag1 (d), Hes1 (e) and Notch1 (f). G: IHC staining of Notch1, Hes1, Jag1 and Hes5 in cerebrum from SAH-induced animal model. (n = 6 for each group; *p < 0.05, **p < 0.01).

SAH mice model was created as previously described in the experimental method. Animal grouping has been described in Figure 1a. The expression of Notch1 signaling pathway proteins was determined within 12 h, 24 h, 48 h, and 72 h after the establishment of SAH through Western blot (Figure 1b), immunohistochemistry was determined at 72 h (Figure 1g) and RT-qPCR (Figure 1c-F) assays. According to the results, the expression of Notch1, Hes 1, Hes 5, and Jag5 were all reduced in a time-dependent manner, with the most significant protein expression inhibition being observed 72 h post SAH. These results exhibit that SAH-induced brain injury is associated with impaired Notch1 signaling.

### 3.2 Jlk6 and si-Notch1 inhibits Notch1

**Figure 2.**
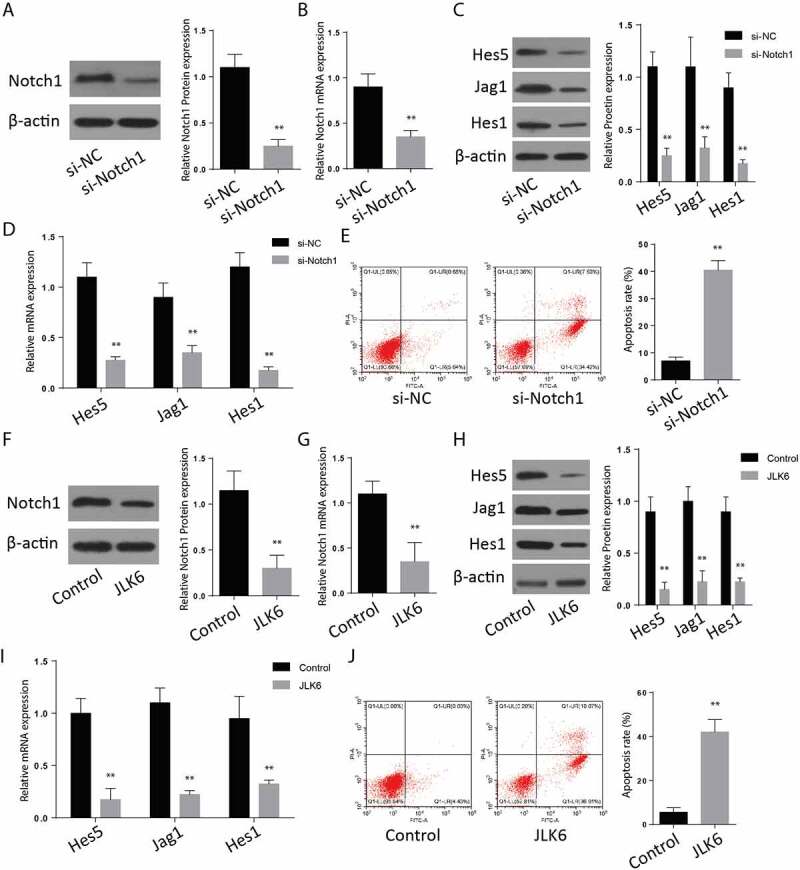
**Downregulation of Notch1 induces apoptosis in-vitro**. A-B: Protein expression (a) and mRNA expression (b) of Notch-1 after transfection of si-Notch1. C-D: Protein expression (c) and mRNA expression (d) of Hes1, Jag1 and Hes5 after transfection of si-Notch1. E: Flow cytometry analysis after transfection of si-Notch1. F-G: Notch1 protein expression (f) and mRNA expression (g)after JLK6 transfection. H-I: Protein expression (h) and mRNA expression (i) of Hes1, Jag1 and Hes5 after JLK6 transfection. J: Flow cytometry analysis after transfection of JLK6. (*p < 0.05, **p < 0.01).

The brain cells were transfected with si-Notch1 plasmids to silence the Notch1 proteins. The expression of Notch1 and the associated proteins on both the control and Si-Notch1 samples was determined through Western blotting to confirm transfection’s successfulness. According to the results, the expression of Notch1 was significantly reduced on the si-Notch 1 samples (Figure 2a). The expression of Notch 1 associated proteins; Jag1, Hes1, and Hes 5, were also significantly repressed following the Notch1 silencing (Figure 2c). RT-qPCR results exhibited same results (Figure 2b-D). Further, apoptotic studies through Annexin V-FITC/PI staining and flow cytometry were performed after silencing. The results indicated an increased number of cells that stained positive for both Annexin V-FITC and PI dyes (34%), which indicated a significantly increased apoptosis. In the control experimental cells, only 4% of cells stained positive for both dyes (Figure 1e).

To investigate the effect of JLK6 on notch1 expression, cells were treated with JLK6, and a Western blot assay was performed to analyze Notch1 expression in the treated and untreated cells. According to the observations, the Notch 1 expression was significantly suppressed in the cells treated with JLK6 (figure 2f). The expression of Notch1 associated proteins including Jag5, Hes1, and Hes 1 were also significantly reduced in the samples treated with the JLK6, Notch1 inhibitor (Figure 2h). RT-qPCR showed similar results (Figure 2g-I). Later, we analyzed for apoptosis in the case of Notch1 inhibition. The results of Annexin V-FITC/PI staining showed an upregulated cells which stained positive for both Annexin V-FITC and PI dyes (34%), which confirmed a rapid occurrence of apoptosis following inhibition of notch1 proteins (Figure 2j). These results showed that inhibition of Notch1 is involved in the upregulation of brain cell apoptosis.

### 3.3 Notch1 inactivation increases apoptotic protein expression and suppresses Bax and cytochrome C

**Figure 3.**
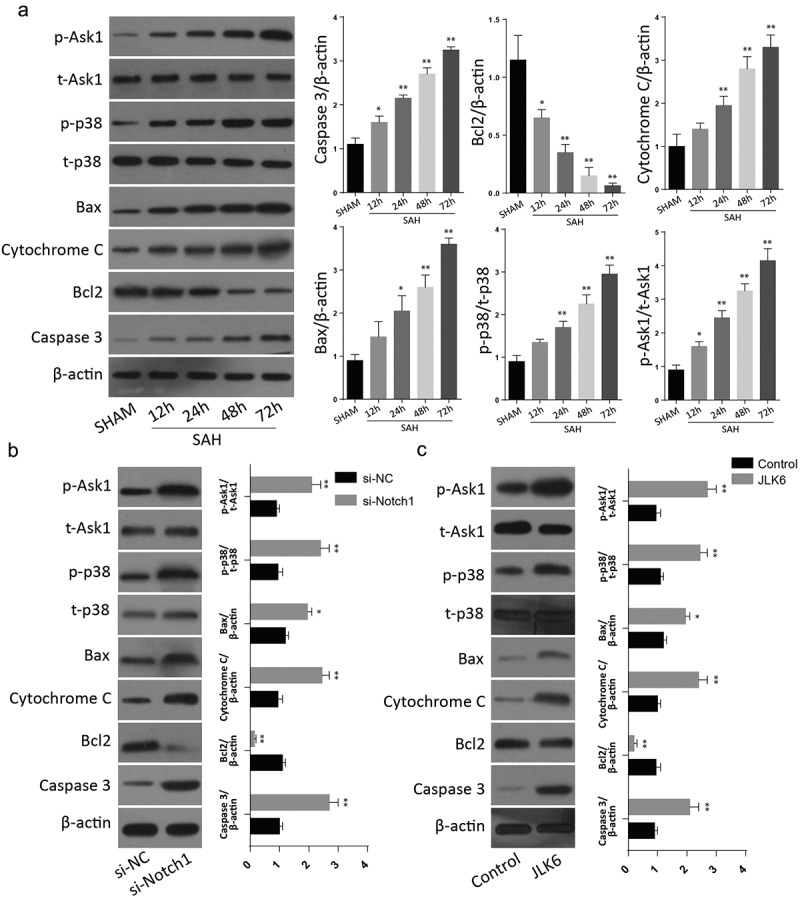
**Silencing of Notch1 regulate the apoptosis through Notch1/ASK1/p38 MAPK signaling pathway**. A: Western blot analysis of Notch1/ASK1/p38 MAPK signaling pathway dependent apoptosis in SAH-induced animal model (n = 6 for each group). B: Western blot analysis of Notch1/ASK1/p38 MAPK signaling pathway dependent apoptosis in si-Notch1 transfection in vitro. C: Western blot analysis of Notch1/ASK1/p38 MAPK signaling pathway dependent apoptosis in JLK6 transfection in vitro. (*p < 0.05, **p < 0.01) .

Further analysis of apoptotic proteins through Western blot after inhibition of notch protein demonstrated a significant upregulation of Caspase 3, cytochrome C and Bax but a significant downregulation of Bcl-2, following notch1 silencing or inhibition. P-ASK1 and p-p38 expressions were also increased following Notch1 inactivation through SAH injury or Notch1 inhibition through si-Notch1 or JLK6 (Figure 3 a&B). These results shows that inhibition of Notch1 is involved in the Ask1/p38/MAPK signaling pathway to regulate the brain cell apoptosis.

### 3.4 Fluoxetine reduces SAH-induced neurologic function impairment

**Figure 4.**
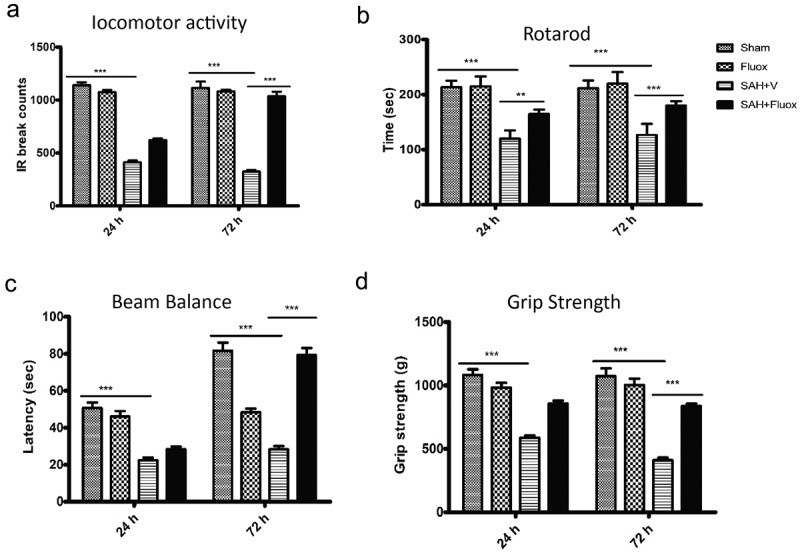
**Fluoxetine reduces SAH-induced neurologic function impairment**. Assessment of neurological function; (a) locomotor activity, (b) rotarod, (c)Beam balance, (d)Grip strength were examined on 24 h and 72 h post-surgery. The SAH mice group demonstrated significant neurological deficits in comparison to the sham group, which was however significantly improved in the SAH + Fluoxetine group (SAH + Fluox). The data are shown as mean± SEM (n = 10 per group). **p < 0.05, *** p < 0.001.

To assess the role of fluoxetine on neuro-protection on SAH, neurological investigations were carried out on 24 h and 72 h after SAH. The observations indicated that locomotor activity in SAH group was significantly reduced in comparison to the sham group after 24 h and 72 h post-SAH (p < 0.001) (Figure 4a). Treatment with Fluoxetine significantly improved the locomotor activity after SAH after 72 h (p < 0.001). SAH induction led to marked impairment in the mice ability of remaining on the rotarod and beam in comparison to the sham group, as shown in Figure 4b. The fluoxetine-treated mice demonstrated remarkably elevated fall latencies in comparison to the SAH mice (Figure 4c; p < 0.001). Further, a significant reduction in the grip strength was observed in the SAH group compared to a sham group (p < 0.001; Figure 4d). The fluoxetine-treated animals indicated a significant increase in the grip strength compared to the SAH group after 72 h (p < 0.001). The impaired steps’ percentage in the sum of steps needed for crossing the tapered beam were also estimated. Finally, the impaired steps were remarkably increased in SAH group than in the sham group after 24 h and 72 h (Figure 4d; p < 0.001). Errors in SAH+ Fluoxetine group were significantly reduced in comparison to those of SAH group after 72 h (p < 0.001). Together, the neuro-behavioral assessment showed that impairment of neurological function induced by SAH was reduced by treatment with Fluoxetine.

### 3.5 Fluoxetine reverses the effects of notch1 inhibition in SAH

**Figure 5.**
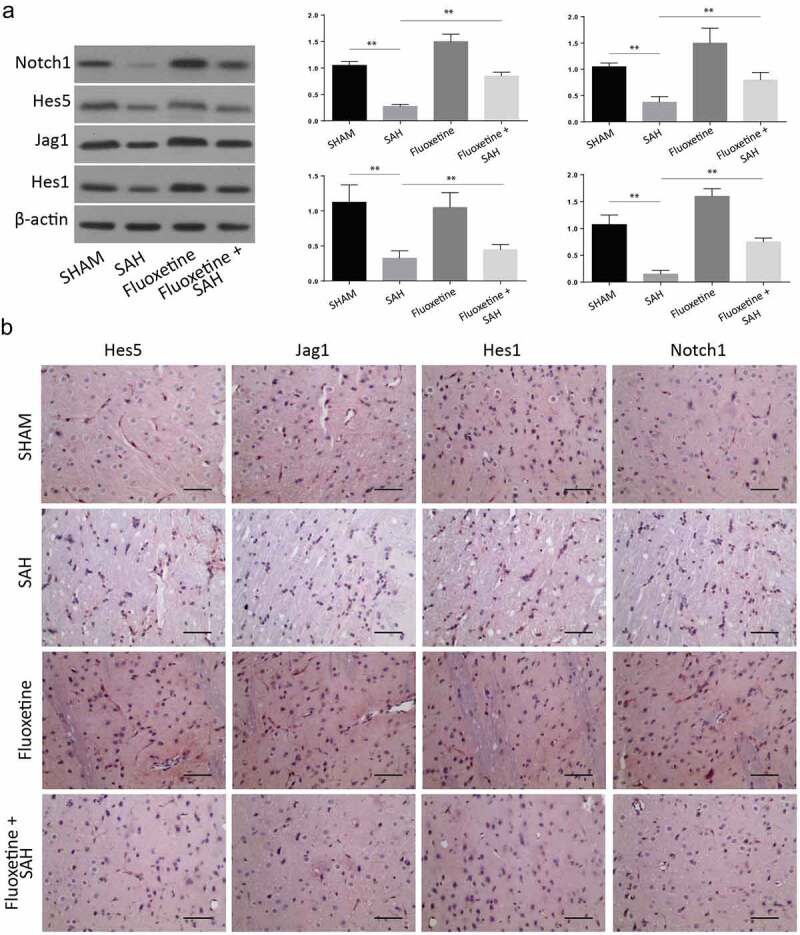
**Delivery of Fluoxetine reduces the Notch1 signaling in SAH-induced animal model**. A: Western blot analysis exhibiting protein expression of Notch1, Hes1, Jag1 and Hes5, following the treatment with fluoxetine. B: IHC staining of Notch1, Hes1, Jag1 and Hes5 in cerebrum from SAH-induced animal model after Fluoxetine treatment. (n = 6 each group; **p < 0.01).

We later aimed at studying the expression of apoptotic proteins following the treatment of SAH with Fluoxetine (serotonin selective reuptake inhibitor). The Western blot analysis confirmed an increase of these proteins following treatment with Fluoxetine compared to the SAH animals (Figure 5a). According to the IHC of the cortex tissue samples, the expression of Notch1 pathway proteins, including Notch1, Hes1, hes5, and Jag5, which had been down-regulated 72 h post-SAH, were reversed, hence significantly increased after Fluoxetine treatment (Figure 5b). These results showed that fluoxetine improved the Notch1 signaling in the SAH-induced brain injury.

### 3.6 Neuroprotective Fluoxetine effects involve apoptosis suppression after SAH

**Figure 6.**
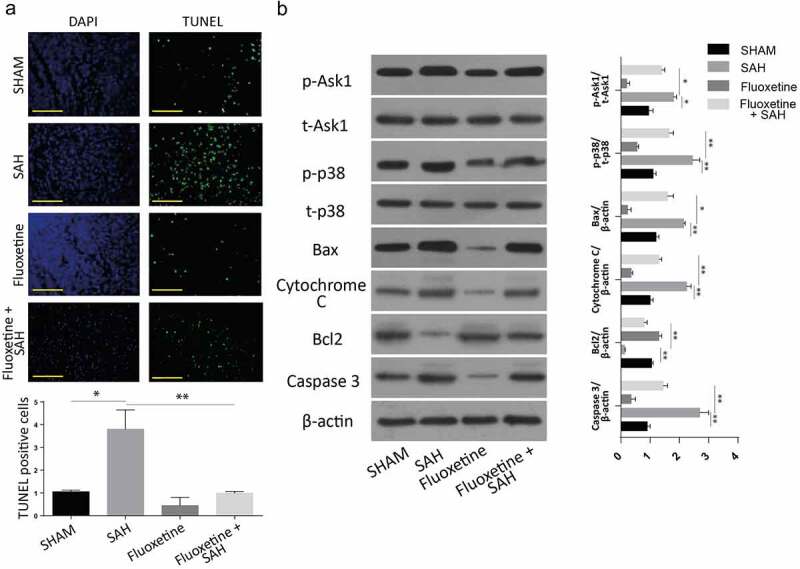
**Delivery of Fluoxetine reduces the apoptosis through Notch1/ASK1/p38 MAPK signaling pathway**. A: TUNEL assay showing the number of apoptotic cells in SAH model after Fluoxetine treatment. B: Western blot analysis of Notch1/ASK1/p38 MAPK signaling pathway dependent apoptosis after delivery of Fluoxetine in SAH-induced animal model. (n = 6 each group; *p < 0.05, **p < 0.01).

To investigate the effects of Fluoxetine on apoptotic protein expression, 10 mg/kg of Fluoxetine was injected intravenously into mice 6 hours after the introduction of SAH. Equal amounts of PBS were injected intravenously into the sham and vehicle mice at similar time points after injury. According to the stained cortex tissue samples results, the SAH mice without Fluoxetine treatment had the highest number of cells that stained positive for TUNEL. However, the number of cells was significantly reduced following treatment with Fluoxetine in all the experimental groups (Figure 6a).

Finally, we also investigated the expression of proteins after the treatment of mice with Fluoxetine. The results also demonstrated an increased caspase 3, BCL-2 apoptotic proteins, and reduced cytochrome c and Bax apoptosis-associated proteins following SAH treatment with Fluoxetine. Similarly, P-ASK1 and P-p38 protein expressions were also significantly downregulated following Fluoxetine treatment (Figure 6b). These results showed Fluoxetine reduced the apoptosis in SAH-induced brain injury through Notch1/Ask1/p38/MAPK signaling pathway.

## 4. Discussion

The EBI mechanism post-SAH is a complex phenomenon involving a sudden intracranial pressure rise and reducing the pressure of cerebral perfusion, necrosis, apoptosis, autophagy, disruption of BBB, inflammation, and oxidative stress [32]. Several molecule effectivities have been investigated on EBI inhibition following SAH, and according to the observations, only agents that target multiple-signaling pathways have provided promising outcomes. The present investigation demonstrated that prophylactic administration of Fluoxetine attenuated EBI after experimental induction of SAH. Fluoxetine administration suppressed apoptosis-associated proteins; thus, the neuroprotective impacts might be essential in its possible anti-apoptosis mechanisms.

The notch signaling pathway is a large transmembrane receptor molecule essential for cell fate and development, proliferation, differentiation, adhesion, and apoptosis [33]. The Notch proteins family is made up of four receptors (Notch1–4) and ligands that comprise of Jagged (Jag1 and Jag2). Compromised Notch-1 expression is associated with some of the cardiovascular or neurodegenerative diseases. During early development of the neurons, Notch is highly expressed in neural precursor cells and plays a critical role in regulating neural proliferation and differentiation. Notch1 is also an essential modulator of neural stem cell maintenance and self-renewal at the developmental stage [34]. According to an in vitro investigation, the expression of Notch1 and its downstream signaling target (Hes1) occur in SVZ cells post-brain stroke [35]. Further works in mice have demonstrated that Notch mutations affect neural plasticity and neuronal development [36].

Additionally, Notch signaling has been confirmed to modulate angiogenesis and the formation of endothelial cells. Related investigations have also found that the levels of Notch1 protein and mRNA are down-regulated in injured tissue, but are then gradually upregulated to the normal levels in the early stage of traumatic brain injury. Similarly, the mRNA levels of the Notch1 ligand, Jagged1 have been reported to be reduced after injury. In a study, where the Notch signaling pathway was controlled using knockdown and overexpression constructs, the Notch signaling pathway activation by Jagged1 or Notch1 overexpression enhanced the invasiveness, migration and angiogenic capability of endothelial progenitor cells. However, the Notch signaling pathway suppression using Jagged1 or Notch1 siRNAs suppressed the migratory capacity, angiogenic and invasive ability of endothelial progenitor cells. The Notch signaling pathway activation in vivo in a mild traumatic brain injury rat model enhanced neurovascular repair, confirming the importance of the Notch signaling pathway activation in blood vessel formation and the repair of tissues after brain trauma [37].

Notch pathway dysregulation has been reported in various neurological conditions, such as ischemia. Notch1 overexpression has specifically been linked with disease severity, and its pharmacological regulation has been reported as a possible therapeutic target [41,42]. In our current findings, Notch1 signaling pathways and their associated proteins, including Hes 1, Hes 5, and Jagged 5, were upregulated following the Fluoxetine treatment. Our observations agreed with the previous report that Jag-dependent signaling of Notch is essential for the proliferation of astrocytes [37, 38, 39, 40]. We believe that it is necessary to develop Notch1 knock-out mice to fully understand the role of Notch1 in SAH.

Cytochrome C, caspase 3, and Bax were involved in apoptosis post-SAH initiation, and beneficial results were obtained when their activities were inhibited during SAH [43]. The current investigation provided evidence that treatment with Fluoxetine down-modulated the expression of cytochrome c and caspase-3, down-regulated cell apoptosis, and ameliorated neurological results at 72 h after SAH. The current findings, taken together, suggested that administration of Fluoxetine, targeting Notch1/ASK1/p38 MAPK signaling pathway may provide a suitable therapeutic modality after SAH.

Despite the numerous studies on SAH, a narrow understanding of the involved mechanisms resulting in early brain injury is currently available. Nonetheless, various reports recently demonstrated apoptosis’s possible central involvement in the pathogenesis of EBI following SAH [44]. Consequently, the cascades of apoptosis provide various possible remedial opportunities that could be used for the treatment of EBI. According to the existing experimental data, the mentioned cascades occur relatively immediately following an initial insult and are directly linked with the SAH-associated physiological abnormality [45]. In agreement with the previous findings [46], our results showed significantly increased TUNEL-positive cell numbers in SAH mice, and Fluoxetine intravenously administered remarkably downregulated the expression of apoptosis post SAH. For successful apoptosis, caspases, a cysteine proteases cascade must be triggered via proteolytic cleavage, which eventually target essential structural and homeostatic molecule, like fodrin and actin and the nuclear protein genes, for instance, poly (ADP-ribose) polymerase (PARP), whose consequence is the fragmentation of DNA and a final cell death. Activation of Caspase-3, Bax, and cytochrome C proteins are considered among the final steps leading to apoptosis [47]. Caspase-3 proteolytically cut various essential cellular molecules that induce the apoptosis-associated changes, such as PARP-1, which remains an essential biochemical apoptosis characteristic. Fluoxetine significantly suppressed the increased expression of caspase-3 protein 72 h after SAH.

Apoptosis signal-regulating kinase 1 (ASK1) is a gene essential for regulating cells’ fate in various diseases, including SAH. It is mitogen-activated protein kinase kinase (MAPKKK and MAP3K), activated by inflammation and oxidative stress [48]. The role of ASK1 in apoptosis has been reported. Induction of the expression of ASK1 expression has been shown to promote apoptotic cell death after ischemia, whereas its silencing improves the brain cerebral infarction [49]. ASK1 activates p38; consequently, treatment of mice with Fluoxetine post-SAH inhibited p-ASK1 and p-p38 to reverse the effects of apoptosis.

## Conclusion

In conclusion, our investigation provided evidence that early Fluoxetine treatment significantly attenuated apoptosis and the expression of apoptosis-related proteins cytochrome C, Bax, and caspase-3 levels after 72 h post-SAH. This investigation provides a novel finding that Fluoxetine may ameliorate early brain injury after subarachnoid hemorrhage through anti-apoptotic effects and Notch1/ASK1/p38 MAPK signaling pathway.

## Highlights

- Subarachnoid hemorrhage (SAH) reduces the Notch1 signaling.
- Inhibition of Notch1 through si-Notch1 or JLK6, specific inhibitor of Notch1, induces the apoptosis through Notch1/ASK1/p38 MAPK signaling pathway.
- Delivery of fluoxetine inhibits the SAH induced apoptosis through Notch1/ASK1/p38 MAPK signaling pathway.
